# Virological footprint of CD4^+^ T-cell responses during chronic hepatitis C virus infection

**DOI:** 10.1099/vir.0.017699-0

**Published:** 2010-06

**Authors:** Vicki M. Fleming, Gillian Harcourt, Eleanor Barnes, Paul Klenerman

**Affiliations:** Peter Medawar Building for Pathogen Research, Nuffield Department of Clinical Medicine, University of Oxford, South Parks Road, Oxford, UK

## Abstract

Human and animal model evidence suggests that CD4^+^ T cells play a critical role in the control of chronic hepatitis C virus (HCV) infection. However, despite their importance, the mechanism behind the failure of such populations in chronic disease is not understood and the contribution of viral mutation is not known. To address this, this study defined the specificity and virological footprint of CD4^+^ T cells in chronic infection. CD8^+^ T-cell-depleted peripheral blood mononuclear cells from 61 HCV genotype 1-infected patients were analysed against a panel of peptides covering the HCV genotype 1 core – a region where CD4^+^ T-cell responses may be reproducibly obtained. In parallel, the core region and E2 protein were sequenced. Gamma interferon-secreting CD4^+^ T-cell responses directed against multiple epitopes were detected in 53 % of individuals, targeting between one and four peptides in the HCV core. Viral sequence evaluation revealed that these CD4^+^ T-cell responses were associated with mutants in 2/21 individuals. In these two cases, the circulating sequence variant was poorly recognized by host CD4^+^ T cells. Bioinformatics analyses revealed no overall evidence of selection in the target epitopes and no differences between the groups with and without detectable CD4^+^ T-cell responses. It was concluded that sustained core peptide-specific CD4^+^ T-cell responses may be reproducibly measured during chronic HCV infection and that immune escape may occur in specific instances. However, overall the virological impact of such responses is limited and other causes for CD4^+^ T-cell failure in HCV must be sought.

## INTRODUCTION

Hepatitis C virus (HCV) is a major cause of liver disease globally (Alter, 2007). The virus is able to evade host innate and adaptive immune responses in immunocompetent adults and to set up persistent infection in the majority of people. Those persistently infected are at risk of progressive liver fibrosis, cirrhosis and cancer. A minority of those infected, however, clear the virus spontaneously, a feature that is reproduced in animal models (Bowen & Walker, 2005a).

The immunological response to HCV has an important bearing not only on the acute outcome (i.e. persistent infection versus spontaneous resolution), but also potentially on the long-term outcome in chronic carriers, although our understanding of the immune responses in acute outcome is more complete. Successful outcome is associated with the maintenance of broadly directed CD4^+^ and CD8^+^ T-cell responses, with maintained functionality (Bowen & Walker, 2005a). Specific HLA class I and II molecules are associated with a successful response (McKiernan *et al.*, 2004; Neumann-Haefelin *et al.*, 2006), and depletion studies in animals strongly support the critical role of both CD4^+^ and CD8^+^ T-cell subsets in acute control (Grakoui *et al.*, 2003; Shoukry *et al.*, 2003).

The responses in those with established persistent infection are typically described as highly attenuated, especially in the blood (Bowen & Walker, 2005a; Klenerman & Hill, 2005; Lauer *et al.*, 2004; Lechner *et al.*, 2000; Rehermann *et al.*, 1996). Chronically infected human and chimpanzee studies based on proliferation responses in blood, or analysis of T-cell lines after stimulation, have typically found either no or only very limited responses (Diepolder *et al.*, 1995; Missale *et al.*, 1996).

In contrast to these studies, recent *ex vivo* analyses based around gamma interferon (IFN-*γ*) secretion have indicated some CD4^+^ T-cell responsiveness in those with persistent infection: we and others have found responses to pools of core peptides in a range of patients with chronic disease, associated with low proliferative capacity and loss of interleukin (IL)-2 secretion (Barnes *et al.*, 2002; Harcourt *et al.*, 2006; Ruys *et al.*, 2008; Semmo *et al.*, 2005, 2007); the responses obtained were reproducible and robust and were not detected in healthy normal controls. Responses using an identical assay against antigens from genotype 1 NS3–NS5b typically yield minimal responses (undetectable or weak) and are difficult to study further. Loss of CD4^+^ T-cell responses to these peptides is seen in the context of human immunodeficiency virus (HIV) infection, associated with an increased viral load (Harcourt *et al.*, 2006).

The mechanism behind the failure of CD4^+^ T-cell responses to contain virus in chronic infection has not been established, nor has the mechanism underlying their low levels in blood (Bowen & Walker, 2005a, b; Klenerman & Hill, 2005). Individual reports have indicated that immune escape may occur in epitopes targeted by HCV-specific CD4^+^ T cells (Eckels *et al.*, 1999, 2000; Puig *et al.*, 2006; Wang & Eckels, 1999; Wang *et al.*, 2003). Variation within epitopes has also been described as affecting T-cell cytokine secretion (Wang & Eckels, 1999; Wang *et al.*, 2003). It is well established that escape from HCV-specific CD8^+^ T cells can occur and may be critical for persistence (Cox *et al.*, 2005b; Dazert *et al.*, 2009; Erickson *et al.*, 2001; Ray *et al.*, 2005; Weiner *et al.*, 1995). However, as CD4^+^ T-cell responses have been considered difficult to detect during chronicity, this issue has not been systematically addressed for T helper populations.

To address this, we analysed CD4^+^ T-cell responses in a large, well-defined cohort of individuals with chronic HCV genotype 1 infection. We systematically analysed the *ex vivo* CD4^+^ T-cell response and defined the fine specificity of this response in relation to the sequence of autologous virus. Surprisingly, we found a persistent IFN-*γ* response in the majority of donors, often targeting multiple epitopes, including a newly defined immunodominant peptide in the core. We evaluated the relationship between T-cell responses targeting individual peptides and the sequence variation within those peptides, and defined the functional consequences of viral mutation and the impact of CD4^+^ T-cell escape in this cohort.

## RESULTS

### *Ex vivo* CD4^+^ T-cell responses are detectable in the majority of donors

In this study, we excluded CD8^+^ T-cell responses at the outset by the use of CD8-depleted peripheral blood mononuclear cells (PBMCs). We measured *ex vivo* responses in an IFN-*γ* enzyme-linked immunosorbent spot (ELISPOT) assay using established robust methods and identified a response against the pool of genotype 1 core peptides in 32/61 genotype 1-positive donors. These donors were collected prospectively and independently of previous immunological or clinical data (Table 1). Alanine transaminase (ALT) levels, viral loads, age and prior treatment status did not differ significantly between the two groups. This frequency of response is very similar to that defined in two previous studies on HCV^+^ chronically infected cohorts using whole undepleted PBMCs and peptide pools (Semmo *et al.*, 2005, 2007).

### Fine specificity of CD4^+^ T-cell responses in persistent infection

**Fig. 1.**
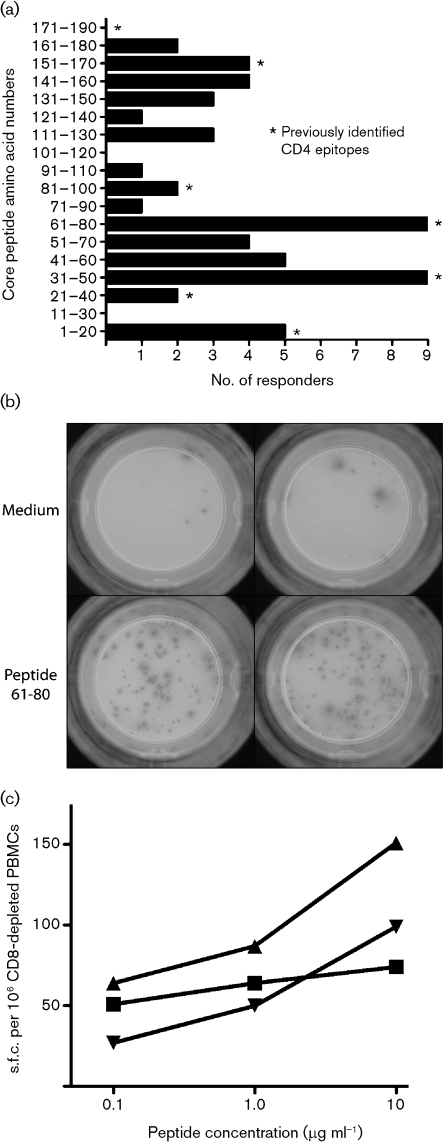
IFN-*γ* ELISPOT response to core peptides. (a) The frequency of CD4^+^ T-cell responses against the panel of core peptides is indicated. Data were derived from Table 2. The sequences of the individual peptides are available in Supplementary Table S1. All donors were tested against all peptides, and the total numbers of donors positive from the 61 tested is shown. (b) Typical ELISPOT assay from patient 306, showing a positive IFN-*γ* response to HCV core peptide 61–80 (s.f.u. per 10^6^ CD8-depleted PBMCs=140). (c) Titration experiment using fresh *ex vivo* CD8-depleted PBMCs from donor 304. The peptide concentration used is displayed on the *y*-axis, with the background subtracted. Peptides: ▴, 31–50; ▪, 61–80; ▾, 151–170.

In this study, we wished initially to define the peptide targets of the observed CD4^+^ T-cell responses. The results of this are illustrated in Table 2 and Fig. 1(a). Table 2 shows the raw data for individual responses to single peptides derived from the core peptide sequence, as well as responses to a pool of the 18 core peptides (core pool). Only data from those responding to one or more peptides are shown (*n*=32). Individuals responded to diverse peptides across the sequence [spot forming cells (s.f.c.) per 10^6^ CD8-depleted PBMCs, mean 170; range 50–732]. If responses to two adjacent peptides were considered as a single target (a conservative estimate), we observed, on average, responses to two epitopes per subject (median 2; 15 subjects with one response, 12 subjects with two responses, four subjects with three responses and one subject with four responses).

The group data are shown in Fig. 1(a). This revealed responses to the majority of peptides tested, with the commonest targets being peptide 31–50 (Lamonaca *et al.*, 1999; MacDonald *et al.*, 2002) and peptide 61–80 (Löhr *et al.*, 1996). Both of these peptides were recognized in 9/32 individuals. The magnitude of the response varied from 50 to 732 s.f.c. per 10^6^ CD8-depleted PBMCs (Table 2) and representative ELISPOT wells are shown in Fig. 1(b). These responses were reproduced in repeat assays, and titrations of the individual peptide, including epitope 61–80, showed that concentrations as low as 1–0.1 μg ml^−1^ were still able to stimulate 50 % maximal responses (Fig. 1c).

### Impact of T-cell responses on viral sequence

We next addressed the relationship between the presence of CD4^+^ T-cell responses and viral sequence evolution. This is important for three reasons: (i) to define to what extent the detectable T-cell responses are targeting autologous sequences; (ii) to define the impact of potential T-cell-mediated selection pressure; and (iii) to analyse whether the non-responder group had accumulated significant mutations as a potential cause of T-cell failure. Sequences were therefore analysed as bulk sequence and, in addition, clonally derived sequences were obtained in a subset of patients.

**Fig. 2.**
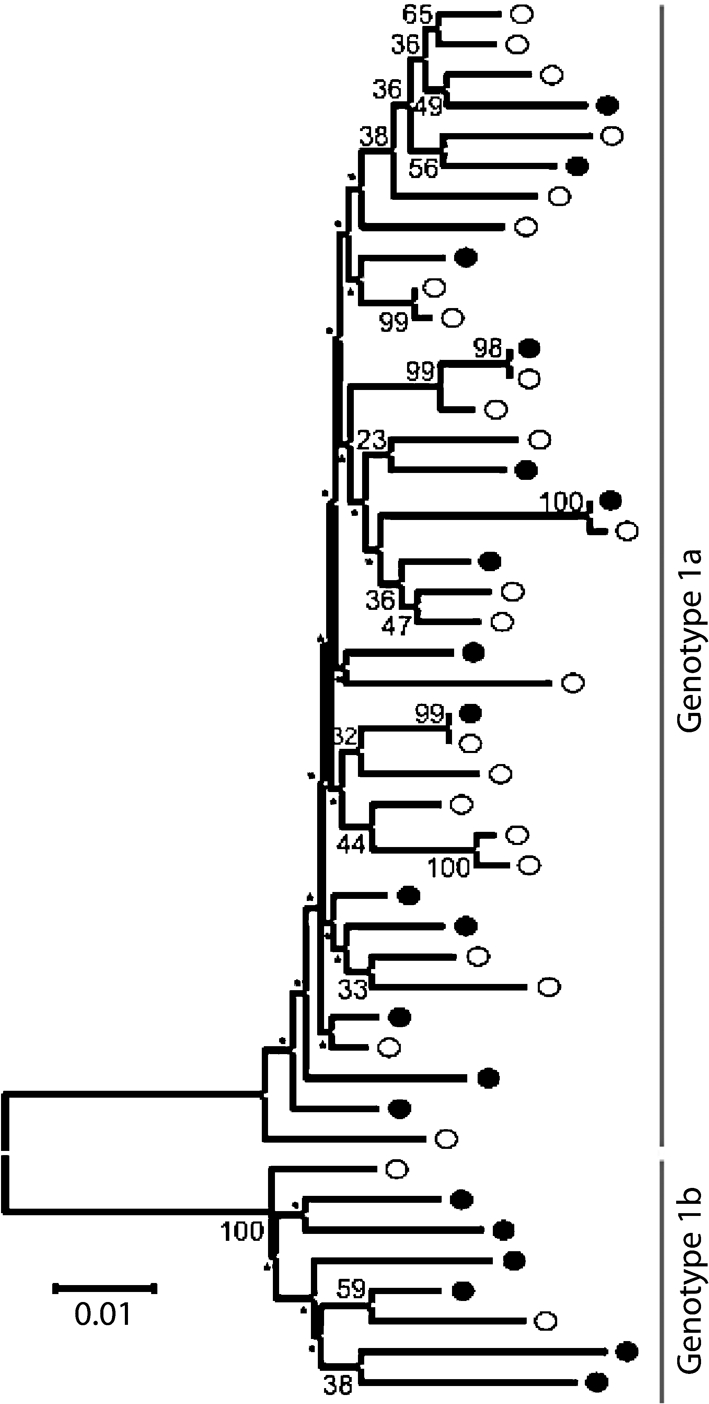
Relatedness of sequences amongst responders and non-responders. Phylogenetic tree of the CD4 responder (•) and non-responder (○) HCV core region based on the neighbour-joining method using 1000 bootstrap replicates (scores <30 are indicated by an asterisk). Bar, nucleotide substitutions per site.

Firstly, we analysed the relatedness of sequences to address whether responder and non-responder sequences clustered separately. Phylogenetic analysis of the core region sequences revealed a typical subdivision into genotype 1a and 1b subtypes (Fig. 2). No correlation was observed between HCV subgenotype and the CD4^+^ T-cell response. In one case, a CD4 responder and non-responder both shared identical core regions, placing them on the same branch of the tree.

**Fig. 3.**
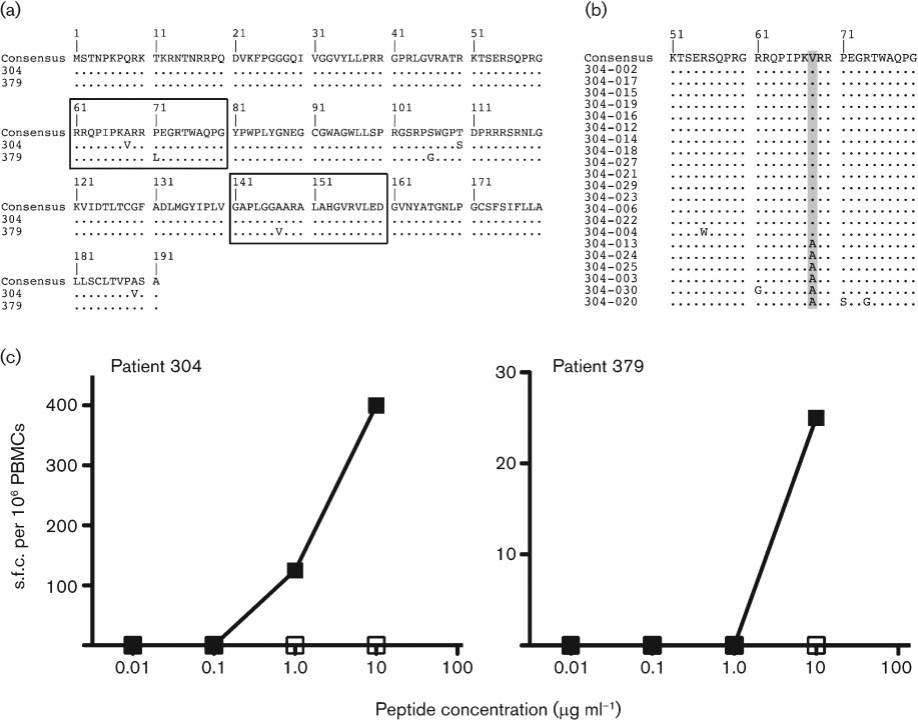
Sequence mutants in targeted epitopes. (a) An alignment of the core region is shown. The upper line indicates the group consensus. The lower lines indicate donors 304 and 379 with mutations within targeted epitopes indicated. Dots indicate amino acids identical to the consensus sequence. (b) An alignment of the core region for cloned donor 304 is shown. Each clone was compared with the bulk sequencing product. The frequency of the variant within the epitope 61–80 is indicated by shading: A68V was observed in the majority of the sequenced population. (c) Peptide titrations using PBMCs from donors 304 and 379, using wild-type (▪) and mutant (□) peptide as indicated in Fig. 3(a). The assays were performed as in Fig. 1.

Forty-six sequences were obtained: 23 CD4 responder core sequences and 23 CD4 non-responder sequences. Amongst the responders, T-cell responses to previously defined peptides were observed in 12 donors and responses to novel peptides (i.e. not previously published as epitopes) in ten donors. From these, all but two donors (304 and 379) showed conserved sequences within the peptides targeted (i.e. identical to the peptides used in the assay). This indicated that the selection of escape mutants through immune pressure from CD4^+^ T cells targeting the HCV core region appears to be rare, at least in this group of individuals. However, single variants were observed within previously defined epitopes, peptide 61–80 (A68V) in one individual and peptide 141–160 (A147V) in another (Fig. 3a). Two additional mutations were observed in both individuals within peptide 101–120 (donor 304: T110S; donor 379: S106G); however, CD4 responses were not detected at this or neighbouring peptides.

To determine the frequency at which these variants were observed within these two donors, the core–E1 region was cloned and sequenced (donor 304: *n*=21 sequences; donor 379: *n*=14 sequences). At aa 68 within donor 304, the mutation to valine was observed in the majority of the cloned population (Fig. 3b; 71.4 %). Within donor 379, the quasispecies population at amino acid position 147 remained identical to the mutant variant valine.

To establish whether T-cell reactivity was linked overall to positive selection within the core, the frequencies of synonymous (dS) and non-synonymous (dN) substitutions and mean pairwise diversity (*π*) were calculated for the CD4 responders and non-responders; these gave an indication of, respectively, the putative levels of selection and nucleotide diversity within each population (Table 3). Patients exhibiting a CD4^+^ T-cell response to the core peptides did not display a greater level of nucleotide diversity or level of selection (*P*>0.05). The values of dS/dN were substantially lower, as expected, in E1 compared with the core, but this did not differ between the two groups. Further analysis identified no evidence for significant positive selection at individual codons within the core region for both CD4 responders and non-responders. Cloning of the core–E1 region for patients 304 and 379 also identified no positively selected sites.

### Functional impact of epitope mutants

To test whether the donor CD4^+^ T cells were able to recognize autologous viral sequence in the two cases where variants were observed (donors 304 and 379), *ex vivo* ELISPOT assays were performed using wild-type and mutant peptides (Fig. 3c). The peptides were titrated to determine the functional avidity of the response. In both cases, there was a clear-cut lack of recognition of the autologous mutant peptide, despite consistent reactivity against the wild-type peptide, suggesting in both cases that CD4^+^ T cells were unable to respond to the endogenous variant.

## DISCUSSION

Virus variation is a major feature of HCV infection, and immune escape from cellular and humoral immune responses is thought to play a significant role in the evolution of chronic infection (Bowen & Walker, 2005a, b; Cox *et al.*, 2005b; Ray *et al.*, 2005). During chronic infection, it is well recognized that there is a loss of CD8^+^ and CD4^+^ reactivity in the blood towards a range of peptides, compared with patients where infection has been resolved (Bowen & Walker, 2005a; Cox *et al.*, 2005a; Day *et al.*, 2003; Lauer *et al.*, 2004; Lucas *et al.*, 2007; Missale *et al.*, 1996; Semmo *et al.*, 2005, 2007). Although a link between immune pressure mediated by CD8^+^ T cells and selection of immune escape variants has been clearly shown (Bowen & Walker, 2005a, b; Cox *et al.*, 2005a), few comparable data exist for CD4^+^ T cells. The impact of virus variation on T-cell function can be profound (Wang & Eckels, 1999), and CD4^+^ escape has been documented in other viral infections (Ciurea *et al.*, 2001), as well as in specific cases in HCV (Puig *et al.*, 2006). However, evaluation of such CD4^+^ T-cell responses in chronic hepatitis C is extremely difficult as they are typically described as ‘weak’ or ‘absent’ in persistent infection. However, the use of conventional assays of T-cell proliferation in these analyses may miss populations of CD4^+^ T cells with different functional profiles. We have recently shown using fresh PBMCs in both ELISPOT assays and a carboxyfluorescein succinimidyl ester proliferative evaluation (Semmo *et al.*, 2005, 2007) that certain CD4 populations are maintained, but that they are low in both IL-2 production and proliferative capacity, features similar to HIV-specific CD4^+^ T-cell populations (Day & Walker, 2003). Assays may also be limited by substantial sequence mismatching between the viral antigens used in the assays and those present in the donor's circulating virus – this matters particularly in cases where superinfection has occurred (Harcourt *et al.*, 2003; Schulze Zur Wiesch *et al.*, 2007).

In this study, we first sought to examine HCV-specific CD4^+^ T-cell responses in a large, carefully selected cohort of chronic HCV patients in order to clearly map the frequency of the peptide-specific response. The HCV genotype 1 core was selected as an appropriate target due to previously identified reproducible responses to this antigen in studies of persistently infected patients (Barnes *et al.*, 2002; Harcourt *et al.*, 2006; Semmo *et al.*, 2005, 2007; Weiner *et al.*, 1995). In addition, the core is a relatively conserved protein and, as a result, sequence variation between the peptides used in the study and the viral strains present in donors should be limited. Peptides were tested individually in order to analyse the breadth of responses and to avoid competitive inhibition. The use of CD8-depleted PBMCs and a sensitive *ex vivo* ELISPOT assay for IFN-*γ* enabled us to detect relatively low-frequency cell populations and those with limited proliferative capacity, and thus provided a specific and accurate picture of the HCV CD4^+^ population in the cohort. Responses to non-structural antigens were very weak in this study, and similar results were also obtained using independently generated overlapping peptide sets spanning the entire genome (data not shown). Although such proteins would represent ideal targets for future studies, the lack of responses to these antigens in *ex vivo* assays severely limits the practicality and scale of such analyses.

HCV core-specific CD4^+^ T cells were readily detectable in just over 50 % of chronically infected patients using the IFN-*γ* ELISPOT assay, similar to results found in other studies (Semmo *et al.*, 2005, 2007). The majority of core peptides were recognized, but responses focused particularly on aa 31–50 and 61–80. Within an individual, up to four different regions were recognized, with the majority of donors recognizing two or more distinct peptides even within this small region of the viral genome. Such data suggest that the level of multi-specificity of *ex vivo* responses in blood during chronic infection may previously have been underestimated. Even our analysis may be an underestimate, as the use of overlapping peptides as described might still fail to detect certain responses. The HLA class II types of those responding to individual peptides were not consistent in each case (data not shown), but data from other studies of virus-specific CD4^+^ T cells have shown that peptides typically bind and are presented by diverse HLA class II molecules (Gerlach *et al.*, 2005; Lamonaca *et al.*, 1999). However, the finding of highly targeted peptides within this conserved region, even though it is an underestimate of the total antiviral CD4^+^ T-cell response, may still provide an important focus in future for analyses of CD4^+^ T-cell responses, e.g. with MHC class II tetramers.

Sequencing of the core region of autologous virus revealed only two mutations within the recognized epitope, indicating that escape from recognition by specific CD4 cells is not a common occurrence. The CD4^+^ T-cell non-responder group acted as important controls in this respect, as it could be argued that the responses are most likely to be sustained in those with intact sequences; however, in the non-responder group, mutation was also rare. Phylogenetic and selection analysis did not resolve the two groups, and no signature of immune selection was detected within the region. Larger studies looking for HLA footprints may be required to define the impact of relatively infrequent selection events (Ray *et al.*, 2005).

In the two cases where mutations did occur, these were novel mutations not found in sequence databases (Combet *et al.*, 2007; Hraber *et al.*, 2007) and both were associated with a functional impact on T-cell recognition. In the absence of the infecting strain, it is not possible to prove that the mutations occurred within the donors. However, in the case of donor 304, infection with HCV occurred as a result of a blood transfusion with no further apparent exposure. This makes it unlikely that superinfection with a mutant strain had occurred, but rather that this change has emerged within the host through immune selection pressures, with two-thirds of the viral clonal population possessing the A68V mutation.

As the core is a conserved viral gene, the failure to generate escape mutants may be related to the fitness cost to the virus of such changes. Interestingly, the A147V mutation lies at the beginning of a structural helix in the lipid droplet-binding domain (D2) of the core. Shavinskaya *et al.* (2007) showed that this amino acid substitution could enhance virus production through improved binding of the core to lipid droplets. Thus, loss of fitness does not seem to be a major argument for conservation of this residue. The impact of viral mutation may be greater on other sites within the core, although escape mutation sites in conserved regions of the core and NS3, which are targeted by CD8^+^ T cells, have been identified (Komatsu *et al.*, 2006; Ray *et al.*, 2005; Timm *et al.*, 2007).

The impact of virus-specific CD4^+^ T cells on virus sequence and vice versa have been addressed in previous studies, although with more limited patient and hence responder numbers. In a single case report, induction of substantial CD4^+^ T-cell responses against HCV in a chimpanzee was associated temporally with the emergence of a mutation that eliminated recognition (Puig *et al.*, 2006). In studies of T-cell lines derived from individuals with chronic infection, it has been observed that mutations can impact on the functional responses of T cells, including a switch away from a Th1 phenotype (Wang & Eckels, 1999). Despite these data, the overall role of virus escape in evasion of CD4^+^ T cells has not been fully addressed. Our data suggest that the phenomenon may occur, but that it is not common, at least among readily detectable responses. A recently performed longitudinal analysis of four infected chimpanzees concurs with this conclusion; these mutations occurred only rarely in this chronically infected HCV model (Fuller *et al.*, 2008).

Exactly what the impact is of such persistent CD4^+^ T-cell responses with intact viral sequences on viral load and liver pathology is still not clear and may demand a larger study, incorporating larger numbers of antigenic targets. Studies to define the impact of target-specific CD8^+^ T-cell responses on HIV viral load (a setting where T-cell responses are much more readily detectable) have required cohorts in the order of 300–400 (Kiepiela *et al.*, 2007). Alternatively, it may be that similar studies of liver-derived cells may be required to define the specificity and magnitude of the responses and their function at the site of infection (Penna *et al.*, 2002; Schirren *et al.*, 2000). Longitudinal studies may also reveal specific mutations, although these patients have been chronically infected for many years and acute infecting sequences are not available. For studies of CD8^+^ T-cell responses, cross-sectional approaches have revealed very obvious ‘footprints’ using a bulk sequencing approach, with similar or even smaller patient numbers, so although there are limitations in the approach used, it can be quite sensitive if selection pressure is strong (Dazert *et al.*, 2009).

In conclusion, we observed sustained CD4^+^ T-cell responses in a large group of individuals persistently infected with HCV genotype 1, which targeted multiple peptides within the core protein. The responses were not associated with a clear virological footprint, and virologically responders and non-responders did not differ significantly, but in those cases where this was observed, mutation had an impact on T-cell recognition. This is inconsistent with a major role for immune escape in impairment of CD4^+^ T-cell responses against HCV, although it may occur in specific instances or in distinct antigenic targets. In this study, we focused on responses to the HCV core in genotype 1; it will be of importance to analyse whether the same process occurs in those responses targeting other viral proteins and/or in other genotypes, which may be under different functional constraints. Similarly, although cross-sectional studies do have significant power to detect escape, sequential studies of patients tracked through acute disease may provide an important alternative strategy. Defining the mechanisms underlying the failure of CD4^+^ T cells to contain HCV and the factors that determine their magnitude and function, longitudinally and especially in the liver, remain important goals in future studies.

## METHODS

### Study subjects.

Sixty-one subjects were included in the study from the Hepatitis Clinic at the John Radcliffe Hospital, Oxford, UK. All patients had persistent genotype 1 HCV infection and consented according to a locally approved protocol (COREC 04.OXA.010). Patients who had received treatment ending within the last 12 months were excluded. Of the 61 patients, 15 had received prior unsuccessful treatment with interferon and/or ribavirin (mean end 4.1 years previously, range 4–14 years). The clinical details of the patients are shown in Table 1.

### T-cell assays

#### IFN-*γ* ELISPOT.

Fresh blood was obtained from the 61 individuals and PBMCs were separated on a density gradient. CD8^+^ cells were depleted using magnetic beads (Dynal) and the CD8^−^ PBMCs assayed in an IFN-*γ* ELISPOT assay (MabTech) using 2×10^5^ cells per well, against a pool of 18 core peptides (10 μg ml^−1^ final concentration for each peptide) to observe overall responses and a panel of 18 individual overlapping 20mer peptides covering the HCV genotype 1 core (aa 1–191; see Supplementary Table S1, available in JGV Online; 10 μg ml^−1^ final concentration) to provide fine detail. In addition, recombinant genotype 1 NS3–NS5 (Chiron; 2 μg ml^−1^), cytomegalovirus-infected cell lysate (Virusys; 2 μg ml^−1^) and phytohaemaglutinin were included as positive controls. Each antigen was tested in duplicate wells and the frequency of IFN-*γ*-producing cells was calculated by subtracting the mean number of s.f.c. per 10^6^ CD8-depleted PBMCs in the negative-control wells (cells/medium alone) from the mean number of s.f.c. in the test wells (Semmo *et al.*, 2005). A positive response was regarded as one in which the difference above the negative-control value of the s.f.c. per well was calculated to be significant (*P*<0.05; Excel binomdist). In specific experiments, a pool of genotype 1-derived NS5A peptides, consisting of 40 overlapping 18mers (BEI Resources) was used.

In further experiments to define the efficacy of the responses or examine the effect of mutations on T-cell recognition, wild-type and/or mutant peptides were tested in serial dilutions in RPMI 1640 in pairs, using the ELISPOT technique as above.

#### HCV sequence evaluation.

Viral RNA was extracted from plasma samples using a Viral RNA Extraction kit (Qiagen). Using a combined reverse transcription and first-round PCR step to amplify the core–E1 region, a 5063 bp external fragment was amplified using 10 pmol of primers utr-246 (5′-GACTGCTAGCCGAGTAGTGTTG-3′) and NS-5315 (5′-CGACCTCYARGTCNGCYCACATRC-3′) (Liu *et al.*, 2004). A second-round PCR was performed with the inner primer utr-277 (5′-CCTTGTGGTACTGCCTGATAG-3′) and a modification of the outer primer C-E1 (5′-GTDGGNGACCARTTCATCATCAT-3′) (Corbet *et al.*, 2003). Using a SuperScript III One-Step RT-PCR kit with Platinum *Taq* DNA polymerase (Invitrogen), RT-PCR cycling conditions were as follows: 55 °C for 30 min and 94 °C for 2 min, followed by 39 cycles of 15 s at 94 °C, 30 s at 57 °C and 5.5 min at 68 °C, with a final extension of 68 °C for 10 min. The inner PCR conditions were as follows: 94 °C for 2 min and ten cycles of 15 s at 94 °C, 30 s at 56 °C and 1 min at 72 °C, followed by 20 cycles of 15 s at 94 °C, 30 s at 56 °C and 1 min increasing by 5 s every cycle at 72 °C, with a final extension of 72 °C for 20 min using a high-fidelity *Taq* DNA polymerase (Expand High Fidelity PCR System; Roche). PCR fragments were gel purified (Qiagen) and the population was sequenced bidirectionally using Prism Big Dye (Applied Biosystems) on an ABI 3100 DNA automated sequencer.

Where necessary, PCR products were also cloned (TOPO TA; Invitrogen) and the DNA purified as above (Qiagen) and sequenced as above. Sequences were edited using Sequencher v4.8 (Gene Codes) and aligned with the Se-Al v2.0 sequence alignment editor (Rambaut, 2007). Kimura's two-parameter model was implemented using mega 4.0 to create neighbour-joining phylogenetic trees (Kimura, 1980). Bootstrap analyses were carried out with 1000 replicates. Levels of dS and dN mutations and *π* were calculated using mega 4.0. To detect evidence of selection at individual codons, single likelihood ancestor counting was used as implemented in DataMonkey (Kosakovsky Pond & Frost, 2005a, b). The analysis was conducted with both the HKY85 and general reversible models of nucleotide substitution with a cut-off *P* value of 0.1.

#### HCV load.

Based on the method outlined by Komurian-Pradel *et al.* (2001), HCV viral load quantification was carried out using real-time PCR with SYBR Green I detection (Roche). The 5′ HCV non-coding region was transcribed into cDNA using primer RC21 (5′-CTCCCGGGGCACTCGCAAGC-3′) (Besnard & Andre, 1994) and following the manufacturer's instructions for SuperScript II Reverse Transcriptase (Invitrogen). Real-time PCR was carried out with 1 μl cDNA with 10 pmol primer RC1 (5′-GTCTAGCCATGGCGTTAGTA-3′) and primer RC21 in a final volume of 25 μl. The reaction was performed in a LightCycler 480 (Roche). The PCR cycling conditions were as follows; an initial denaturation step at 95 °C for 15 min, followed by 45 cycles of 95 °C for 30 s, 60 °C for 30 s and 72 °C for 15 s. For each step, the temperature transition rate varied between 2.2 and 4.4 °C s^−1^, with fluorescence measurements taken after each elongation step. Conversion of copies ml^−1^ to IU ml^−1^ was performed using the HCV RNA genotype panel (National Institute for Biological Standards and Control, UK).

#### Statistical analysis.

Levels of ALT, viral load and T-cell responses were compared using the Mann–Whitney test. Pearson's chi-squared test was used to compare genetic diversity between groups for the core and E1. *P*<0.05 was considered statistically significant. Statistics were analysed using Prism V (Graphpad Software) and Excel (Microsoft).

## Supplementary Material

[Supplementary Table]

## Figures and Tables

**Table 1.** Clinical features of the study cohort na, Not available.

**Patient no.**	**M/F**	**Age (years)**	**ALT level (U l^−1^)**	**Viral load (IU ml^−1^)**	**Core response**	**Core region sequenced?**
106	F	71	135	110 925	Positive	Yes
112	M	53	62	5 017	Positive	Yes
117	M	45	39	61 929 806	Positive	Yes
141	M	28	31	26 183	Positive	Yes
160	M	50	34	na	Positive	No
168	M	59	40	17 913	Negative	Yes
171	M	45	39	51 739	Negative	No
172	M	50	38	478 192	Positive	Yes
182	F	52	38	71 729	Negative	Yes
183	M	48	199	2 920 108	Negative	No
188	F	48	29	7 172 883	Negative	Yes
191	M	56	168	na	Negative	No
193	M	46	103	685 931	Positive	Yes
201	M	52	78	235 960	Positive	Yes
205	M	56	111	56 642	Negative	Yes
208	M	28	59	52 131	Negative	Yes
210	M	43	443	5 056 294	Negative	No
215	M	49	77	1 046 535	Negative	Yes
252	M	44	17	92 895	Negative	Yes
263	F	50	36	999 500	Positive	No
269	M	73	26	489 951	Negative	Yes
287	M	54	32	1 152 365	Negative	Yes
289	F	49	25	960 304	Negative	No
303	M	45	34	27 681	Positive	Yes
304	F	47	24	111 709	Positive	Yes
305	F	41	67	132 091	Negative	Yes
306	M	54	56	89 367	Positive	No
308	F	62	33	10 504 549	Positive	No
310	F	50	186	169 719	Positive	Yes
311	F	47	47	1 226 837	Positive	Yes
317	F	46	34	15 992	Positive	Yes
318	F	42	21	603 620	Negative	Yes
319	M	61	327	486 031	Negative	Yes
320	F	50	15	9 995	Positive	Yes
323	F	44	50	127 425	Positive	Yes
324	F	40	76	333 559	Positive	Yes
328	M	38	96	36 139	Negative	Yes
338	M	47	94	97 990	Positive	Yes
342	F	40	33	93 679	Negative	Yes
343	M	47	57	86 231	Positive	Yes
348	M	27	51	11 092 491	Negative	Yes
349	M	52	40	23 674	Negative	Yes
350	F	71	81	82 704	Positive	Yes
365	F	55	115	175 206	Positive	Yes
369	M	55	91	148 553	Negative	Yes
370	M	45	101	33 669	Positive	No
371	F	40	58	1 046 535	Negative	Yes
375	F	59	130	228 121	Positive	Yes
376	M	58	134	41 940	Positive	No
379	M	50	47	226 553	Positive	Yes
382	F	54	115	153 649	Negative	Yes
384	M	52	40	212 051	Negative	Yes
386	M	45	85	635	Positive	No
388	M	36	12	5 997 000	Negative	Yes
393	M	54	230	587 941	Negative	Yes
396	M	47	24	438 996	Positive	Yes
401	M	49	50	3 484 532	Positive	Yes
402	M	40	167	na	Positive	No
403	F	52	115	na	Negative	No
404	M	53	34	na	Positive	No
405	M	57	27	na	Negative	No

**Table 2.** HCV core CD4^+^ IFN-*γ* ELISPOT responses of 32 positive subjects Results are given as s.f.c. per 10^6^ CD8-depleted PBMCs.

**Patient no.**	**Core pool**	**Peptide**																		**No. of epitopes**
		**1–20**	**11–30**	**21–40**	**31–50**	**41–60**	**51–70**	**61–80**	**71–90**	**81–100**	**91–110**	**101–120**	**111–131**	**121–140**	**131–150**	**141–160**	**151–170**	**161–180**	**171–190**	
106	195					190														1
112	160	135																		1
117	283				150															1
141	60									65										1
160	250														120		120			2
172	250	280						175						150						3
193	56					350	560													2
201	100							320												1
263	350							170												1
303	100														65			60		2
304	297			77	110		77	220									173			3
306	870					313		140												2
308	140				124	159														1
310	169												97		150					2
311	114				198															1
317	291				80			80		90								127		4
320	166							155									145			2
323	60				182															1
324	110														80					1
338	335												180				268			2
343	137						168													1
350	120								125											1
365	250	405		175	732			254												3
370	105															70				1
375	185	128						85												2
376	772	675																		1
379	294				109											80				2
386	50				50															1
396	100												66			50				2
401	100							150							80					2
402	250							110	90				125							3
404	85			90	95															2

**Table 3.** Genetic diversity of the HCV core and E1 in CD4^+^ T-cell responders and non-responders

	**Core**		**E1**	
	**dS/dN**	***π***	**dS/dN**	***π***
All individuals (*n*=46)	26.86	0.048	8.13	0.149
CD4^+^ responders (*n*=23)	26.88	0.055	9.23	0.167
CD4^+^ non-responders (*n*=23)	25.83	0.041	6.93	0.132
