# Supplementary material for: Virological footprint of CD4+ T-cell responses during chronic hepatitis C virus infection
Source: J Gen Virol. 2010 Jun;91(Pt 6):1396–406. doi: 10.1099/vir.0.017699-0 (PMC3052717; doi:10.1099/vir.0.017699-0)
Supplement: [Supplementary Table] [file supp_91_6_1396__index.html]

 Virological footprint of CD4+ T-cell responses during chronic hepatitis C virus infection -- Fleming et al. 91 (6): 1396 Data Supplement - Supplementary Table -- Journal of General Virology

### Virological footprint of CD4+ T-cell responses during chronic hepatitis C virus infection, by V. M. Fleming, G. Harcourt, E. Barnes and P. Klenerman

*Journal of General Virology* vol. **91**, part 6, pp. 1396 –1406

  

**Supplementary Table S1.** HCV core peptide amino acid sequences [PDF] (38 KB)

  
  
