# Supplementary material for: Virological footprint of CD4+ T-cell responses during chronic hepatitis C virus infection
Source: J Gen Virol. 2010 Jun;91(Pt 6):1396–406. doi: 10.1099/vir.0.017699-0 (PMC3052717; doi:10.1099/vir.0.017699-0)
Supplement: [Supplementary Table] [file supp_91_6_1396__1.pdf]

Supplementary Table S1. HCV core peptide amino acid sequences

No.	Amino acid range	Sequence (5′→3′)
1	1–20	MSTNPKPQRKTKRNTNRRPQ
2	11–30	TKRNTNRRPQDVKFPGGGQI
3	21–40	DVKFPGGGQIVGGVYLLPRR
4	31–50	VGGVYLLPRRGPRLGVRATR
5	41–60	GPRLGVRATRKTSERSQPRG
6	51–70	KTSERSQPRGRRQPIPKARR
7	61–80	RRQPIPKARRPEGRTWAQPG
8	71–90	PEGRTWAQPGYPWPLYGNEG
9	81–100	YPWPLYGNEGCGWAGWLLSP
10	91–110	CGWAGWLLSPRGSRPSWGPT
11	101–120	RGSRPSWGPTDPRRRSRNLG
12	111–130	DPRRRSRNLGKVIDTLTCGF
13	121–140	KVIDTLTCGFADLMGYIPLV
14	131–150	ADLMGYIPLVGAPLGGAARA
15	141–160	GAPLGGAARALAHGVRVLED
16	151–170	LAHGVRVLEDGVNYATGNLP
17	161–180	GVNYATGNLPGCSFSIFLLA
18	171–190	GCSFSIFLLALLSCLTVPAS

Fleming, V. M., Harcourt, G., Barnes, E. & Klenerman, P. (2010). Virological footprint of $CD4^+$ T-cell responses during chronic hepatitis C virus infection. *J Gen Virol* **91**, 1396–1406.
